# Performance of Clinical and Laparoscopic Criteria for the Diagnosis of Upper Genital Tract Infection

**DOI:** 10.1155/S1064744997000501

**Published:** 1997

**Authors:** Jeffrey F. Peipert, Lori A. Boardman, C. James Sung

**Affiliations:** 1 Division of Ambulatory Care, Department of Obstetrics and Gynecology Women and Infants' Hospital Brown University School of Medicine 101 Dudley Street Providence RI 02905 USA; 2 Department of Pathology Women and Infants' Hospital Brown University School of Medicine Providence RI USA

## Abstract

*Objective:* The purpose of this study was to validate the standard minimal clinical criteria and the laparoscopic triad of tubal edema, erythema, and purulent exudate used to diagnose acute upper genital tract infection.

*Methods:* Subjects included women who either met the Centers for Disease Control and Prevention's (CDC) minimal criteria for acute pelvic inflammatory disease or had other signs of upper genital tract infection (i.e., atypical pelvic pain, abnormal uterine bleeding, or cervicitis). The subjects were evaluated with a baseline interview comprehensive laboratory testing, and either an endometrial biopsy or laparoscopy with endometrial and fimbrial biopsies for definitive diagnosis of upper genital tract infection. Patients were considered positive for upper genital tract infection if they had any of the following findings: 1) histologic evidence of endometritis or salpingitis; 2) laparoscopic visualization of purulent exudate in the pelvis without another source; or 3) positive testing for *Neisseria gonorrhoeae* or *Chlamydia trachomatis* from the endometrium, fallopian tubes, or pelvis.

*Results:* One hundred twenty-nine women with adequate endometrial samples were evaluated between August 1993 and September 1997, and 62 had complete laparoscopic evaluations. The sensitivities of the CDC's minimal clinical criteria for pelvic inflammatory disease and the laparoscopic triad of edema, erythema, and purulent exudate were 65% and 60%, respectively.

*Conclusions:* Commonly used minimal clinical criteria for pelvic inflammatory disease and the laparoscopic triad of tubal edema, erythema, and purulent exudate have limited sensitivity with correspondingly high false negative rates.


Infectious Diseases in Obstetrics and Gynecology 5:291–296 (1997)
© 1998 Wiley-Liss, Inc.

### Performance of Clinical and Laparoscopic Criteria for the Diagnosis of Upper Genital Tract Infection

Jeffrey F. Peipert,[1]* Lori A. Boardman,[1] and C. James Sung[2]

[1]*Division of Ambulatory Care, Department of Obstetrics and Gynecology, Women and Infants' Hospital, Brown University School of Medicine, Providence, RI*

[2]*Department of Pathology, Women and Infants' Hospital, Brown University School of Medicine, Providence, RI*

### Abstract

*Objective:* The purpose of this study was to validate the standard minimal clinical criteria and the laparoscopic triad of tubal edema, erythema, and purulent exudate used to diagnose acute upper genital tract infection.

*Methods:* Subjects included women who either met the Centers for Disease Control and Prevention's (CDC) minimal criteria for acute pelvic inflammatory disease or had other signs of upper genital tract infection (i.e., atypical pelvic pain, abnormal uterine bleeding, or cervicitis). The subjects were evaluated with a baseline interview, comprehensive laboratory testing, and either an endometrial biopsy or laparoscopy with endometrial and fimbrial biopsies for definitive diagnosis of upper genital tract infection. Patients were considered positive for upper genital tract infection if they had any of the following findings: 1) histologic evidence of endometritis or salpingitis; 2) laparoscopic visualization of purulent exudate in the pelvis without another source; or 3) positive testing for *Neisseria gonorrhoeae* or *Chlamydia trachomatis* from the endometrium, fallopian tubes, or pelvis.

*Results:* One hundred twenty-nine women with adequate endometrial samples were evaluated between August 1993 and September 1997, and 62 had complete laparoscopic evaluations. The sensitivities of the CDC's minimal clinical criteria for pelvic inflammatory disease and the laparoscopic triad of edema, erythema, and purulent exudate were 65% and 60%, respectively.

*Conclusions:* Commonly used minimal clinical criteria for pelvic inflammatory disease and the laparoscopic triad of tubal edema, erythema, and purulent exudate have limited sensitivity with correspondingly high false negative rates. Infect. Dis. Obstet. Gynecol. 5:291–296, 1997.
© 1998 Wiley-Liss, Inc.

Key words
adnexitis; endometritis; salpingitis; pelvic inflammatory disease; diagnosis

Upper genital tract infection, commonly referred to as pelvic inflammatory disease (PID), is often a difficult diagnosis to make. Accurate diagnosis is extremely important for the management of the acute disease and the prevention of long-term morbidity. The Centers for Disease Control and Prevention (CDC)[1] suggests that empiric treatment should be instituted on the basis of the presence of abdominal tenderness, adenxal tenderness, and cervical motion tenderness. Only

Presented in part at the Annual Meeting of the Infectious Diseases Society in Obstetrics and Gynecology, Monterey, CA, 1994.

Contract grant sponsor: American Association for AIDS Research; Contract grant number: 02110-15-RG; Contract grant sponsor: Rhode Island Foundation.

*Correspondence to: Dr. Jeffrey F. Peipert, Department of Obstetrics and Gynecology, Women and Infants' Hospital, 101 Dudley Street, Providence, RI 02905. E-mail: jpeipert@wihri

Received 18 April 1997
Accepted 22 September 1997
Clinical Study recently has an attempt been made to validate the minimal criteria, and in this report the sensitivity of these criteria was quite low (33%).[2]

Laparoscopic visualization of tubal edema, erythema, and purulent exudate is considered the "gold standard" for the diagnosis of acute PID.[3,4] However, the accuracy of laparoscopy for the diagnosis of acute PID has been challenged. Sellors et al.[5] demonstrated that laparoscopic visualization has a sensitivity of 50% and a specificity of approximately 85% compared to diagnosis by fimbrial minibiopsy. These findings have resulted in some investigators questioning whether laparoscopy should still be considered the "gold standard" for the diagnosis of PID.

The purpose of this study is to prospectively validate the CDC's minimal clinical criteria for the diagnosis of PID and the laparoscopic triad of tubal edema, erythema, and purulent exudate compared to predefined objective criteria for upper genital tract infection. Diagnostic test characteristics are calculated for the minimal clinical criteria, independent clinical signs, individual laparoscopic findings, and laparoscopic triad of edema, erythema, and purulent exudate.

While infection of the uterus, fallopian tubes, ovaries, and pelvis is commonly referred to as acute PID, others consider acute PID only synonymous with acute salpingitis. To avoid this controversy, the term upper genital tract infection will be used throughout this manuscript.

### SUBJECTS AND METHODS

A cross-sectional study was performed in order to evaluate currently available diagnostic testing and criteria for the diagnosis of acute upper genital tract infection. The methodology for this study has been reported previously.[6] Informed consent was obtained from women who chose to participate in the study if they presented with either "classic" or "non-classic" signs of upper genital tract infection (defined below). We invited consecutive women identified with signs or symptoms of upper genital tract infection to participate. Patients were recruited from an urgent care unit of a large urban women's hospital and referred from adjacent clinics and practices in the hospital vicinity.

Patients with "classic" signs of upper genital tract infection presented with pelvic pain (less than 30 days duration) and were found to have the three CDC stated criteria of abdominal, cervical motion, and adnexal tenderness. The second group included women presenting with "non-classic" signs of upper genital tract infection. These signs included atypical pelvic pain (i.e., pain less than 30 days duration that did not meet the CDC's minimal criteria for acute PID), abnormal uterine bleeding of unclear etiology (in women less than the age of 40 years to avoid those who were perimenopausal) or cervicitis as demonstrated by a mucopurulent cervical discharge or a positive cervical microbiologic test for *N. gonorrhoeae* and/or *C. trachomatis*. The choice of this broad range of signs of upper genital tract infection was deliberate: the intention was to include a full spectrum of cases required to evaluate the chosen diagnostic tests.[7,8]

All participants were between the ages of 16 and 45 years; women less than age 18 years were included if they were legally able to consent or if informed consent could be obtained from a legal guardian. Exclusion criteria included pregnancy, delivery, or operative procedure within the preceding 4 weeks, inability to give informed consent, or a history of hysterectomy or bilateral salpingectomy. Women with tubal ligation were not excluded.

Evaluation began with a comprehensive enrollment interview reviewing past medical, obstetrical, and gynecological and sexual history. Physical examination and laboratory testing including complete blood count, erythrocyte sedimentation rate, and C-reactive protein were then performed. Abdominal and pelvic tenderness were recorded and graded based on a modification of the McCormack et al.[9] scale. Vaginal secretions were screened for white blood cells and evaluated for evidence of vaginitis as documented by the presence of clue cells, trichomonads, and pseudohyphae, or spores.

Cervical cultures were obtained for *N. gonorrhoeae* and *C. trachomatis*. Polymerase chain reaction (PCR) testing was also obtained for *C. trachomatis*. Endometrial biopsies were sent for the same cultures as well as for aerobic and anaerobic bacteria. All participants underwent either endometrial biopsy alone or a combination of endometrial biopsy and laparoscopy in order to obtain objective evidence of acute upper genital tract infection. The choice of either a laparoscopic evaluation or an endometrial biopsy alone was made by the patient. While all participants with pain or tenderness were offered a laparoscopic evaluation, subjects without these findings were not offered this option as a laparoscopy was not felt to be clinically indicated. Although we recognized that this two-tiered evaluation process could introduce a selection or detection bias, we felt it was necessary to capture the wide spectrum of upper tract infections.

If laparoscopy was performed, tubal and peritoneal cultures along with a fimbrial minibiopsy were added to the above microbiologic studies. If the fallopian tubes were occluded and appeared distended, then an aspiration of the tubes was performed to identify purulent material and to obtain a specimen for microbiologic evaluation.

We used objective criteria for our outcome of upper genital tract infection. These criteria included any of the following: 1) histologic evidence of acute endometritis or salpingitis; 2) laparoscopic evidence of salpingitis as demonstrated by purulent exudate in the pelvis with no other source; or 3) microbiologic evidence of *N. gonorrhoeae* or *C. trachomatis* from the endometrium, fallopian tubes, or pelvis. For acute endometritis to be verified, neutrophils had to be present in one or more of the following areas in the absence of tissue disintegration such as menstrual changes: the surface epithelium, the subepithelial stroma, and/or the endometrial glands. Although not part of our objective criteria for acute upper genital tract infection, we also evaluated whether using chronic endometritis would change our results. Chronic endometritis was defined by the presence of plasma cells with or without stromal fibrosis. Acute or chronic salpingitis was defined as the presence of neutrophils or plasma cells in the lamina propria of the tubal mucosa. The mere presence of inflammatory cells in engorged blood vessels would be considered negative.[10]

In addition to our objective definition of upper genital tract infection, we performed additional analyses of the subset of women who were found to have laparoscopic or histologic evidence of salpingitis. In this subgroup, we include women with the laparoscopic triad of edema, erythema, and purulent exudate, visualization of a tubo-ovarian abscess, or histologic evidence of acute or chronic salpingitis.

The study protocol was approved by the Women and Infants' Hospital Institutional Review Board prior to initiation of the research. Statistical analysis was performed using $\chi^2$ amd Fisher exact tests for categorical variables, unpaired t-tests for continuous variables, and non-parametric tests for continuous variables that were not normally distributed. $P < 0.05$ was considered statistically significant. Sensitivity and specificity were calculated. Since predictive values are so dependent on the prevalence of the disease in the population, we used Bayes' theorem to calculate predictive values and based these calculations on prevalence rates of 30% and 60%. Separate analyses were performed including women with chronic endometritis to see if the results would vary. Statistical Analysis Software (SAS Institute, Cary, NC) was used for the analysis.

### RESULTS

Between August 1993 and September 1997, 132 women were evaluated for evidence of upper genital tract infection. Three women were excluded due to inadequate endometrial samples. Sixty-five of the 129 patients (52%) underwent an endometrial biopsy and cultures, while 62 (48%) had cultures, endometrial biopsy, and laparoscopy.

The demographic characteristics of the entire study population were as follows: median age = 24 years (range 16–45 years), median gravidity = 2 (range 0–12), median years of education = 12 (range 3–16 years). The median number of sexual partners in the 6 months prior to study inclusion was 1 with a range of 0–5, and the median number of lifetime sexual partners was 4 with a range from 1 to >100. There were no significant differences in age, gravidity, education, race, insurance or marital status, smoking history, or history of sexually transmitted diseases or PID between patients who underwent laparoscopic evaluations and those who were evaluated with endometrial biopsy alone. As expected, participants who met the minimal clinical criteria for the diagnosis of PID were more likely to undergo laparoscopic evaluation than women with "non-classic" presentations ($P = 0.001$).

Of the 70 women who met the minimal criteria for PID, 40% were found to have objective evidence of acute upper genital tract infection. Of the 43 participants with objective evidence of upper genital tract infection, 28 met the minimal criteria for PID (sensitivity = 65%). Of the 59 women with "non-classic" signs of upper genital tract infection, 25% were found to have objective evidence of up-

**TABLE 1. Objective evidence of upper genital tract infection (UGTI) by indication for evaluation**

| Indication | No. evaluated | No. positive | % Positive |
|---|---|---|---|
| Minimal CDC criteria for acute PID (i.e., "classic" UGTI) | 70 | 28 | 40 |
| "Non-classic" UGTI | 59 | 14 | 25 |
| Atypical pain | 32 | 7 | 22 |
| Cervicitis | 12 | 4 | 33 |
| Abnormal bleeding | 13 | 2 | 15 |
| Abnormal bleeding and cervicitis | 2 | 2 | 100 |

per genital tract infection. Table 1 shows the results of the evaluations by indication for enrollment. Six of the 14 women (43%) with evidence of mucopurulent cervical discharge or a positive cervical test for *N. gonorrhoeae* or *C. trachomatis,* but who did not meet the CDC's minimal criteria for PID, were found to have objective evidence of upper genital tract infection.

The diagnostic test characteristics and predictive values of the clinical and laparoscopic findings based on prevalences of 30% and 60% were calculated (Table 2). The most sensitive physical examination finding was the presence of any pelvic tenderness (91%). The minimal clinical criteria for the diagnosis of PID had a sensitivity of 65% and specificity of 51%. In the 62 evaluable patients with complete laparoscopic evaluation, the laparoscopic triad of edema, erythema, and purulent exudate had a sensitivity of 60% and a specificity of 100%. If any one of these findings was noted at the time of the laparoscopy, the sensitivity was 100% and specificity was 84%. Separate analyses were performed using chronic endometritis as a positive indicator of upper genital tract infection. The results varied only slightly (±6%).

There were 48 patients who had a laparoscopic evaluation, but did not have the classic triad of edema, erythema, and purulent exudate. Eight (17%) were found to have histologic evidence of acute or chronic endometritis. Two of these 48 patients (4%) had microbiologic evidence of chlamydial infection in the upper genital tract. Forty of the 48 patients had an adequate fimbrial biopsy. Four of 40 patients (10%) without the laparoscopic triad had histologic evidence of salpingitis.

Of the 18 patients with either histologic or laparoscopic evidence of salpingitis (laparoscopic triad or tubo-ovarian abscess), 6 (33%) had normal endometrial biopsies. Four of 9 patients with histologic evidence of salpingitis did not have the classic laparoscopic triad of edema, erythema, and purulent exudate. Of these 4 patients, 2 had erythema alone, 1 had edema and erythema but no purulent exudate, and 1 had erythema and purulent exudate but no tubal edema.

### DISCUSSION

Clinical accuracy in the diagnosis of such upper genital tract infections is currently imprecise; numerous reports comparing clinical and laparoscopic diagnoses of pelvic inflammatory disease have demonstrated the lack of correlation between the two methods.[9,11,12] In this study, we have attempted to validate the accepted clinical criteria and the classic laparoscopic criteria for PID. A clinical research dilemma is how to validate what is currently accepted as the "gold standard" (i.e., laparoscopic visualization). We did this by creating objective criteria that would be maximally sensitive and reflect several measures of infection: histology, microbiology, and visualization of infected structures (laparoscopy).

We found the sensitivities of both the accepted clinical criteria and the triad of laparoscopy visualization of edema, erythema, and purulent exudate to be disappointingly low. Our findings are in agreement with those of Sellors et al.[5] and Korn et al.[2] In a study of 95 women who presented to primary care physicians with pelvic pain, Sellors et al.[5] noted that clinicians had a 41–61% sensitivity for the diagnosis of PID. Laparoscopic diagnosis of PID had a sensitivity of 48–50%. Korn and colleagues[2] noted a 33% sensitivity for the CDC's minimal criteria. Korn et al.,[2] however, used plasma cell endometritis as the objective criterion for upper genital tract infection. It is clear that many cases of upper tract infection will be missed (false negative) if we rely on the strict clinical criteria or the laparoscopic triad of edema, erythema, and purulent exudate.

Our study is one of a few in the medical literature to evaluate "non-classic" signs of PID (atypical pain, abnormal bleeding, positive tests for *N. gonorrhoeae* or *C. trachomatis,* or evidence of mucopurulent cervicitis) in the inclusion criteria. Twenty-five percent of these women with "non-

TABLE 2. Diagnostic test characteristics of clinical and laparoscopic findings for the diagnosis of acute UGTI[a]

| Test | Sensitivity (%) | Specificity (%) | Prevalence = 30% (%) | | Prevalence = 60% (%) | |
|---|---|---|---|---|---|---|
| | | | PPV | NPV | PPV | NPV |
| Entire cohort (N = 129) | | | | | | |
| Clinical criteria | 65 | 51 | 36 | 77 | 66 | 49 |
| Individual examination findings | | | | | | |
| Abdominal tenderness | 81 | 31 | | | | |
| CMT | 67 | 47 | | | | |
| Uterine tenderness | 84 | 32 | | | | |
| Adnexal tenderness | 91 | 23 | | | | |
| Any pelvic tenderness | 91 | 20 | | | | |
| Laparoscopy group (N = 62) | | | | | | |
| Laparoscopic triad | 60 | 100 | 100 | 85 | 100 | 63 |
| Individual laparoscopic findings | | | | | | |
| Edema | 80 | 95 | | | | |
| Erythema | 80 | 86 | | | | |
| Purulent exudate | 80 | 100 | | | | |
| Any of above | 100 | 84 | | | | |

[a]PPV = positive predictive value; NPV = negative predictive value; CMT = cervical motion tenderness.

classic" presentations were found to have objective evidence of upper tract infection.

There are important implications of these findings. If we are interested in maximizing sensitivity (i.e., detect as many cases as possible) to help prevent adverse reproductive sequelae, then clinicians will need to consider "non-classic" presentations, such as pelvic tenderness without the chief complaint of pain ("atypical PID"), pain and pelvic tenderness that do not meet the CDC's minimal criteria, and other clinical signs that are potential indicators of upper genital tract infection (abnormal uterine bleeding, evidence of cervical infection, vaginal discharge, or dysuria). We found the presence of any pelvic tenderness to have the highest sensitivity for the diagnosis of upper genital tract infection. Clinicians should consider empiric treatment of all women with any pelvic organ tenderness and signs of lower genital tract infection such as mucopurulent cervicitis or leukorrhea.

In patients who have a laparoscopy performed for uncertain diagnosis, clinicians should consider empirical treatment of all women with any abnormal laparoscopy finding (edema, erythema, or purulent exudate), rather than focus on the laparoscopic triad as the "gold standard." In fact, a strong argument could be made for treating all women undergoing laparoscopy for the diagnosis of PID since some patients with negative laparoscopic evaluations will have endometritis on biopsy or a positive test for *N. gonorrhoeae* or *C. trachomatis*.

Our study is not without limitations. Ideally, all women enrolled in the study should have complete diagnostic evaluations with laparoscopy and fimbrial minibiopsies. Otherwise, it is possible to have a detection bias in favor of the complete evaluation group. However, we did not feel this could be justified in women without pain or tenderness. In addition, clinicians performing the diagnostic laparoscopy were not blinded to the presentation of the patient. Evidence of upper tract infection, however, was based on objective data which included histology, microbiology, and visualization of purulent exudate. There is no clear explanation why the rate of cervicitis was so low in this population. Perhaps the broad entry criteria resulted in many more cases of symptomatic women with other disease processes rather than a sexually transmitted infection.

Clinical criteria and, in the case of PID, laparoscopic criteria should ideally be validated in a prospective fashion prior to utilization in clinical practice. In this study, both were found to have relatively low sensitivities for the diagnosis of acute upper genital tract infection. Future longitudinal studies should attempt to determine which criteria best correlate with adverse reproductive sequelae.

### REFERENCES

1. Centers for Disease Control and Prevention: 1993 sexually transmitted diseases treatment guidelines. MMWR 42(RR-14):75–78, 1993.

2. Korn AP, Hessol N, Padian N, Bolan G, Muzsnai D, Donegan E, et al.: Commonly used diagnostic criteria for pelvic inflammatory disease have poor sensitivity for plasma cell endometritis. Sex Transm Dis 22:335–341, 1995.
3. Jacobson L, Westrom L: Objectivized diagnosis of acute pelvic inflammatory disease. Am J Obstet Gynecol 105: 1088–1098, 1969.
4. Method MW: Laparoscopy in the diagnosis of pelvic inflammatory disease: Selection criteria. J Reprod Med 33:901–906, 1988.
5. Sellors J, Mahony J, Goldsmith C, et al.: The accuracy of clinical findings and laparoscopy in pelvic inflammatory disease. Am J Obstet Gynecol 164:113–120, 1991.
6. Peipert JF, Boardman L, Hogan JW, Sung J, Mayer KH: Laboratory evaluation of acute upper genital tract infection. Obstet Gynecol 87:730–736, 1996.
7. Ransohoff DF, Feinstein AR: Problems of spectrum and bias in evaluating the efficacy of diagnostic tests. N Engl J Med 199:926–930, 1978.
8. Peipert JF, Sweeney PJ: Diagnostic testing in obstetrics and gynecology: A clinician's guide. Obstet Gynecol 82: 619–623, 1993.
9. McCormack WM, Nowroozi K, Alpert G, et al.: Acute pelvic inflammatory disease: Characteristics of patients with gonococcal and non-gonococcal infection and evaluation of their response to treatment with aqueous penicillin G and spectinomycin hydrochloride. Sex Transm Dis 4:125–131, 1977.
10. Kurman R: Blaustein's Pathology of the Female Genital Tract. 4th ed. New York: Springer-Verlag, pp 534–538, 1994.
11. Jacobson L: Laparoscopy in the diagnosis of acute salpingitis. Acta Obstet Gynecol Scand 43:160–174, 1964.
12. Hadgu A, Westrom L, Brooks CA, Reynolds GH, Thompson SE: Predicting acute pelvic inflammatory disease: A multivariate analysis. Am J Obstet Gynecol 155:954–960, 1986.
